# Synergistic activity of mupirocin in combination with protocatechuic acid ethyl ester against mupirocin-resistant MRSA

**DOI:** 10.3389/fcimb.2026.1722886

**Published:** 2026-02-27

**Authors:** Li Shen, Jinjin Yang, Zhixuan Chen, Jiana Fu, Huilin Zhao, Jianbo Lv, Yu Huang, Xinru Yuan, Haojin Gao, Fangyou Yu, Chunchan Lin, Jingyi Yu

**Affiliations:** 1 Department of Clinical Laboratory, Shanghai Pulmonary Hospital, School of Medicine, Tongji University, Shanghai, China; 2 Department of Laboratory Medicine, The First Affiliated Hospital of Wenzhou Medical University, Wenzhou, China

**Keywords:** antibiotic resistance, mupirocin, protocatechuic acid ethyl ester, *Staphylococcus aureus*, synergistic activity

## Abstract

The prevalence of mupirocin resistance in MRSA severely limits therapeutic options for skin and soft tissue infections. This study aimed to evaluate the potential synergistic activity between mupirocin and protocatechuic acid ethyl ester (EDHB) through *in vitro* and *in vivo* investigations. Clinical *S. aureus* isolates were characterized for antibiotic resistance profiles and molecular features via antimicrobial susceptibility testing, MLST, and *spa* typing. For MRSA isolates, checkerboard and time-kill assays were performed to assess *in vitro* synergy. The potential interference of EDHB with bacterial membrane integrity and efflux pumps was investigated using propidium iodide and ethidium bromide, respectively. The disk diffusion method was applied to test the retained antimicrobial activity of mupirocin and EDHB in ointment formulations. A murine dermal wound model was established to evaluate *in vivo* efficacy by topical application of mupirocin and EDHB, alone or in combination, on infected wounds. EDHB alone exhibited limited activity but synergistically reduced mupirocin MICs by 4-8-fold in most strains. Checkerboard analysis revealed synergistic or partial synergistic interactions against MRSA. Time-kill curves further indicated that combining these two drugs can effectively inhibit the planktonic *S. aureus*. EDHB rapidly disrupts cytoplasmic membrane integrity via concentration-dependent propidium iodide influx, independent of *norA*/*mepA* efflux pump modulation. The enhanced antibacterial activity of mupirocin and EDHB was sustained in ointment formulations, resulting in superior therapeutic outcomes with combination therapy compared to monotherapy. EDHB acts as a membrane-disrupting adjuvant that synergizes with mupirocin against MuR-MRSA, offering a promising strategy to combat recalcitrant *S. aureus* infections through localized combination therapy.

## Introduction

*Staphylococcus aureus* (*S. aureus*) causes diverse infections ranging from minor skin infections to post-operative wound infections (Deurenberg and Stobberingh, 2008). Its remarkable antibiotic adaptability led to the emergence of methicillin-resistant *S. aureus* (MRSA) in the early 1960s (Lee et al., 2018). MRSA has become a major public health concern (Vestergaard et al., 2019) due to its high morbidity and mortality rates (Cosgrove et al., 2003), coupled with resistance to all penicillins and most β-lactam antibiotics (except ceftaroline and ceftobiprole) (Lakhundi and Zhang, 2018). Resistance in multidrug-resistant (MDR) bacteria is mediated by the efflux pumps (Huang et al., 2004; Hou et al., 2023). NorA is one of the primary multidrug resistance efflux pumps (MDR EPs) in *S. aureus (*Ferreira et al., 2022). As a membrane protein, NorA actively extrudes a diverse array of antimicrobial agents, including hydrophilic fluoroquinolones, dyes like ethidium bromide, and biocides such as quaternary ammonium compounds (Yoshida et al., 1990; Costa et al., 2013). Additionally, overexpression of MepA, a protein exhibiting significant residue identity to NorA, has been shown to reduce susceptibility to mupirocin (Mup) in *S. aureus* strains (Huang et al., 2004).

Topical drug delivery has emerged as the most common method for treating skin infections, enabling site-specific pharmacotherapy while lowering systemic adverse effects (Williamson et al., 2017; Benson et al., 2019). Current clinical formulations (e.g., creams, gels, and ointments) achieve precise drug localization through stratum corneum penetration modulation (Barnes et al., 2021). Antibacterial agents are among the most frequently used and even abused drugs for treating various bacterial infections (Leekha et al., 2011; Aldhubiab et al., 2024). Mupirocin is one of the most effective topical antibacterial agents, particularly for treating skin and soft tissue infections (SSTIs), such as impetigo or folliculitis (Lamb, 1991; Gangwar et al., 2021). The effect of mupirocin manifests rapidly, typically within 2–3 days (Sun et al., 2024). It exhibits selective activity against Gram-positive bacteria, particularly staphylococci and streptococci, with negligible efficacy against Gram-negative bacteria (Ward and Campoli-Richards, 1986). Mupirocin is a bacteriostatic agent that inhibits protein synthesis through targeting isoleucyl-tRNA synthetase (IleRS) (Hetem and Bonten, 2013). The emergence of mupirocin resistance presents a substantial challenge in clinical settings, predominantly due to target-based mechanisms, including nonsynonymous mutations in the *ileS* gene or the acquisition of plasmid-borne genes such as *mupA* or *mupB (*Seah et al., 2012). Increased usage has been linked to the rise of mupirocin resistance, with reports indicating that mupirocin resistance can be as high as 81% (Poovelikunnel et al., 2015). This resistance crisis has intensified the exploration of combinatorial strategies to potentiate antibacterial efficacy while circumventing resistance mechanisms, as the overall effectiveness of these treatments is often greater than the sum of their individual effects. Using combination therapy exerts resistance-preventive efficacy by enhancing the pharmacodynamic activity of at least one agent, thereby suppressing the emergence of dominant resistant subpopulations that drive therapeutic failure (Leekha et al., 2011). Numerous studies have shown that natural compounds combined with commonly used antibacterial drugs may offer a new strategy to combat infections caused by multidrug-resistant bacteria (Miklasińska et al., 2015).

Protocatechuic acid ethyl ester (ethyl-3,4-dihydroxybenzoate, EDHB) is an esterified derivative of phenolic acids and is widely distributed in fruits and vegetables, such as peanut seed coats and tea leaves, etc (Miklasińska et al., 2015). It exhibits strong antioxidant activity (Huang et al., 2003; Chu et al., 2010) and can serve as a fat and cream antioxidant, a food additive, and a pharmaceutical intermediate (Li et al., 2016). Previous studies have proved that EDHB has an antibacterial property on *S. aureus (*Miklasińska et al., 2015), but it has not been extensively studied yet. Moreover, EDHB displays significant synergistic activity when combined with clindamycin, and it also demonstrates a certain degree of synergy with erythromycin and vancomycin against certain strains; however, no observable synergistic interaction was detected when combined with cefoxitin (Miklasińska et al., 2015; Miklasinska-Majdanik et al., 2022). Previous studies have evidenced that EDHB enhances antibiotic activity against drug-resistant *E. coli* by acting as a potential efflux pump inhibitor (EPI), without affecting bacterial membrane permeability (Lu et al., 2022). It remains to be explored whether the synergistic interaction arises from Mup-induced upregulation of *norA* and *mepA (*Truong-Bolduc et al., 2022), while EDHB concurrently counteracts it by blocking pump-mediated drug extrusion. Beyond this, it may exert antibacterial effects through disruption of the cytoplasmic membrane of *S. aureus* cells, suggesting a potential mechanism underlying its efficacy. Thus, the research on the antibacterial properties of EDHB is promising and may also confer an additive antimicrobial advantage to other antibiotics.

This study systematically evaluates *in vitro* and *in vivo* antibacterial activity of Mup alone and in combination with EDHB, addressing a critical knowledge gap in dual-target approaches against Mup-resistant *S. aureus* skin infections. The Mup-EDHB combination demonstrates pronounced synergistic effects against mupirocin-resistant MRSA (MuR-MRSA) isolates. Furthermore, this dual-agent system can be effectively formulated into an ointment, highlighting its potential for clinical translation in combating antibiotic-resistant infections while shortening treatment duration and resistance development.

## Materials and methods

### Bacterial strains

Clinical MRSA isolates (n=20) were isolated from three cities (Sichuan, Wuhan, and Guangzhou) in China. Among these strains, 11 strains were isolated from sputum, while the remaining 9 strains were obtained from pus (n=6) and blood specimens (n=3) (**Table 1**). All strains were stored in trypticase soy broth (TSB) containing 30% v/v glycerol at -80 °C. ATCC 29213 served as the quality control for susceptibility testing following the CLSI and EUCAST guidelines, with a well-characterized genome and stable phenotype ensuring result reproducibility. An MSSA strain was used as the quality control instead of an MRSA strain because there is currently no universally recognized MRSA quality control strain, and the inherent *mecA*-mediated resistance in MRSA could interfere with membrane permeability studies, potentially introducing unwanted variability.

**Table 1:** Characteristics of bacterial strains used in this study.

Strain	MLST	*spa*	SCC*mec*	Region	Specimen
MR196	ST-239	t030	IIIa	Sichuan	Sputum
MR209	ST-239	t030	IIIa	Sichuan	Sputum
MR324	ST-5	t2460	IIa	Wuhan	Sputum
MR366	ST-5	t2460	IIa	Wuhan	Blood
MR490	ST-5	t2460	IIa	Wuhan	Blood
MR326	ST-764	t1084	IIa	Wuhan	Sputum
MR390	ST-764	t1084	IIa	Guangzhou	Pus
MR391	ST-764	t1084	IIa	Guangzhou	Sputum
MR398	ST-764	t1084	IIa	Guangzhou	Blood
MR421	ST-764	t1084	IIa	Guangzhou	Sputum
MR422	ST-764	t1084	IIa	Guangzhou	Pus
MR429	ST-764	t1084	IIa	Guangzhou	Pus
MR444	ST-764	t1084	IIa	Guangzhou	Pus
MR445	ST-764	t1084	IIa	Guangzhou	Pus
MR454	ST-764	t1084	IIa	Guangzhou	Sputum
MR460	ST-764	t1084	IIa	Guangzhou	Sputum
MR473	ST-764	t1084	IIa	Guangzhou	Pus
MR475	ST-764	t1084	IIa	Guangzhou	Sputum
MR401	Unknown	t1084	IIa	Guangzhou	Sputum
MR404	Unknown	t1084	IIa	Guangzhou	Sputum

### Antimicrobial agents

Mupirocin (Mup), protocatechuic acid ethyl ester (EDHB), PEG400, and PEG3350 were purchased from MedChemExpress (MCE). Stock solutions of Mup and EDHB were prepared in DMSO at 100 mg/mL. The ointment base was formulated by combining PEG400 (70%, wt/vol) and PEG3350 (30%, wt/vol). For therapeutic formulations, Mup (1%, wt/vol), EDHB (1%, wt/vol), or their combination was homogenously blended into the PEG matrix. Vehicle control containing an equivalent concentration of DMSO in the PEG ointment base was prepared to account for solvent effects. All formulations were freshly prepared, shielded from light, and stored at 4 °C.

### Susceptibility testing

Antimicrobial susceptibility testing was performed according to CLSI guidelines using the broth microdilution method. Minimum inhibitory concentrations (MICs) were determined in duplicate with an initial inoculum of 5×10^5^ CFU/mL in 200 μL cation-adjusted Mueller-Hinton broth (CAMHB) in the presence of Mup or EDHB. Following 16–20 h incubation at 37°C, the MIC was read and defined as the lowest drug concentration that completely inhibited visible growth. Mup susceptibility breakpoints were classified as follows: susceptible (<4 μg/mL), low-level resistance (8-256 μg/mL), and high-level resistance (>512 μg/mL), based on established criteria (Khoshnood et al., 2019).

### Checkerboard assay

The synergistic activity of Mup and EDHB was evaluated using a checkerboard assay. Two-dimensional dilution series (64 combinations) of Mup and EDHB were tested against *S. aureus* (5×10^5^ CFU/mL) in 96-well microtiter plates. After inoculation, the plates were incubated for 16–20 h at 37 °C. Fractional inhibitory concentration index (FICI) was calculated as: FICI=(MIC_combination A_/MIC_alone A_)+(MIC_combination B_/MIC_alone B_), where synergistic activity was defined as FICI≤0.5; partial synergistic activity as 0.5<FICI<1; additive activity as FICI=1, irrelevant activity as 1<FICI≤4, and antagonistic activity as FICI>4 (Ni et al., 2021).

### Time-kill assay

We randomly selected 6 strains as experimental *S. aureus* strains. The overnight cultures were adjusted to 1×10^8^ CFU/mL, and 1:200 diluted with fresh TSB containing Mup, EDHB, or a Mup plus EDHB. Each experiment has a non-medicated bacterial-containing medium as a control. Aliquots were collected at 0, 2, 4, 6, 8, and 24 h, serially diluted 10-fold in sterile PBS. 10 μL of the diluted bacterial solution was uniformly applied to tryptic soy agar (TSA) plates. Colonies were enumerated after overnight incubation. Synergy was identified when the combination achieved a ≥2-log_10_ CFU/mL reduction at 24 h over the most active single agent (Laplante, 2007).

### Membrane integrity analysis using propidium iodide

To characterize the lysis activity of EDHB against bacterial cell membranes, we evaluated the permeability of the membrane using a propidium iodide (PI) fluorescence assay. *S. aureus* cultures were grown in TSB to mid-exponential phase (OD_600_ = 1.0), harvested by centrifugation, and resuspended in PBS to an OD_600_ of approximate 0.7. Bacterial suspensions were supplemented with PI at a final concentration of 10 μg/mL, and 200 μL aliquots were transferred to black 96-well plates. Following 6 min equilibration, serially diluted EDHB was injected into wells. Fluorescence measurements (excitation: 535 nm, emission: 617 nm) were kinetically monitored at 2-min intervals over a 6-min pre-treatment period using a fluorescence microplate reader to establish baseline membrane integrity prior to EDHB addition.

### Efflux pump inhibition analysis using ethidium bromide and mupirocin

To assess the potential of EDHB as an efflux pump inhibitor, the MICs of ethidium bromide (EtBr) and mupirocin (Mup) were determined with or without EDHB co-treatment, using carbonyl cyanide 3-chlorophenylhydrazone (CCCP) as a positive control inhibitor. Bacterial suspensions were standardized to 0.5 McFarland standard in normal saline, diluted 1:100 in brain heart infusion broth (BHI), and aliquoted (100 µL/well) into 96-well plates. Serial dilutions of EtBr or Mup solutions (100 µL) were combined with equal volumes of EDHB (100 µL). Following 24 h static incubation at 37 °C, MICs were recorded as the lowest drug concentration achieving complete visible growth suppression. A reduced MIC of EtBr or specific antibiotics against efflux pump-expressing strains is characteristic of efflux pump inhibition (Dos Santos et al., 2018).

### Real-time fluorescence quantitative PCR

Following 6 h of EDHB exposure, *S. aureus* bacterial cells were harvested and processed for RT-qPCR analysis as described previously (Shen et al., 2023). Total RNA was isolated, reverse-transcribed into cDNA, and the expression levels of *norA* and *mepA* genes were quantified by normalizing to the housekeeping gene *gyrB*.

### *In vitro* ointment antibacterial testing

The *in vitro* antibacterial activity of the tested ointment formulations was evaluated using the disk diffusion method to determine the zone of inhibition. *S. aureus* strain MR366 was cultured overnight and adjusted to a turbidity of 0.5 McFarland standard. Subsequently, the suspension was evenly spread onto Mueller-Hinton agar (MHA) plates to ensure thorough coverage. A central well (4 mm diameter) was aseptically excised from the agar. Next, 40 μL of the test ointments was dispensed into the well. After incubation for 16–20 h, the inhibition zones were visually assessed, and their diameters (including the well diameter) were measured in millimeters using an vernier caliper.

### *In vivo* ointment therapeutic testing

Ointments were tested for *in vivo* antimicrobial effectiveness using a dermal wound model. 6-week-old female BALB/c mice (n=6) were anesthetized via intraperitoneal injection of 100 μL 1.5% pentobarbital. A full-thickness excisional wound was created on the depilated dorsal surface under aseptic conditions using a sterile 6-mm biopsy punch. The wound was inoculated with 10 μL of the exponential stage of *S. aureus* strain MR366 suspension containing 1×10^7^ CFU. After 40 min postinoculation, wounds were topically treated with either monotherapy ointment or combination drug ointment. Treatments were repeated at 12 h intervals over a 48 h period, resulting in four total applications. To monitoring of the healing process, the wound contraction rate is calculated using the following formula, expressed as the percentage reduction in wound area: WC_d_=(1-WA_d_/WA_0_)×100 (Blanchard et al., 2015). Mice were euthanized by cervical dislocation, and the wound tissue was aseptically excised and homogenized in 1 mL sterile PBS. Serial 10-fold dilutions of the homogenate were plated onto TSA plates. After 16 h of incubation at 37 °C, *S. aureus* colonies were counted to quantify bacterial load.

### Statistical analysis

The comparative efficacy of Mup monotherapy versus Mup-EDHB combination therapy against *S. aureus* viability was analyzed using an unpaired Student’s t-test. Differences were considered statistically significant when P<0.05. Analyses were performed using GraphPad Prism (v9.0), with results presented as mean ± standard deviation (SD) of triplicate biological replicates.

## Results

### Antibacterial activities on *S. aureus*

All 20 tested isolates were confirmed as methicillin-resistant *S. aureus* (MRSA) through *mecA* gene detection (**Table 1**). Two SCC*mec* types (IIa and IIIa) were identified among these MRSA strains. The majority of MRSA strains belonged to SCC*mec* IIa (90.00%, 18/20), and the others were SCC*mec* IIIa (10.00%, 2/20). Multilocus sequence typing (MLST) and *spa* typing revealed ST764-t1084 as the predominant lineage (65.00%, 13/20), followed by ST5-t2460 (15.00%, 3/20), ST239-t030 (10.00%, 2/20), and unknown (10.00%, 2/20). The MIC values for Mup ranged from 2 to 512 μg/mL, with 18 strains (90.00%, 18/20) classified as Mup-resistant (MIC ≥8 μg/mL). High-level (0.06%, 1/18) and low-level (94.40%, 17/18) Mup resistance were in these MuR-MRSA isolates. In contrast, EDHB demonstrated consistently higher MIC values across all isolates (512-1024 μg/mL), suggesting limited intrinsic antibacterial activity against MRSA at tested concentrations.

### Synergy testing by checkerboard and time-kill assays

**Figure 1:**
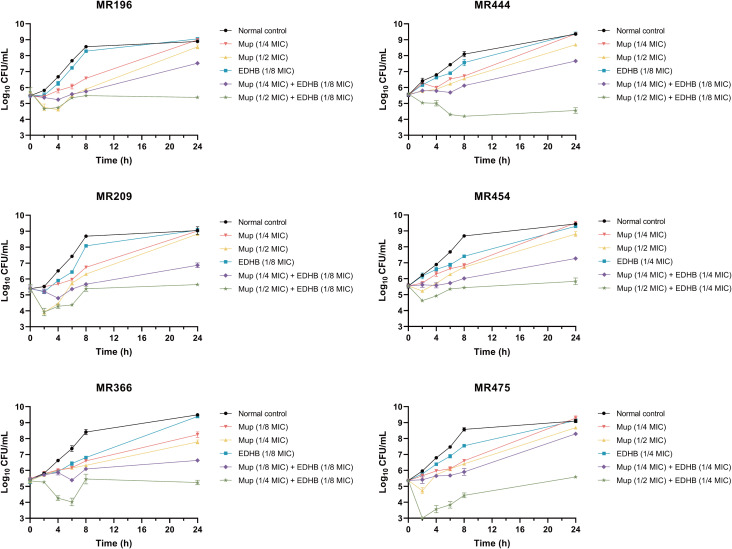
Time-kill curves of MRSA strains in presence of Mup and EDHB alone or in combination, and a normal control (only medium without any drug) for 24 h.

As shown in **Table 2**, synergistic effects (13 isolates) and partial synergistic effects (7 isolates) were observed through checkerboard testing with the Mup and EDHB combination. The addition of EDHB enhanced the sensitivity of Mup by reducing the MIC of Mup by 4- to 8-fold, indicating that the combination of EDHB and Mup exhibited synergistic activity. To further elucidate the combined effects, a 24-hour time-kill assay involving 6 MuR-MRSA isolates was used to observe the changes in the actual viable cell counts of the bacteria after exposure to Mup/EDHB alone or Mup+EDHB (**Figure 1**). While monotherapy with either Mup or EDHB exerted bacteriostatic activity compared to the control group, the antimicrobial efficacy diminished over time, with all MuR-MRSA reaching or approaching untreated growth levels by 24 h. **Table 3** shows that in the 1/4 MIC Mup+EDHB group, only MR209 and MR454 showed >2-log_10_ CFU/mL reduction in viable cells compared to monotherapy. The 1/2 MIC Mup+EDHB combination demonstrated sustained antibacterial effects during early treatment phases, achieving >2.5-log_10_ CFU/mL reductions versus monotherapy at 24 h. Notably, EDHB significantly potentiated the antibacterial activity of Mup, with synergistic effects observed even in highly Mup-resistant *S. aureus* strains. These results implied that the Mup-EDHB combination substantially enhances antimicrobial efficacy.

**Table 2:** MICs and FICIs of Mup combined with EDHB against MRSA.

	MIC (μg/mL) of alone		MIC (μg/mL) of combination			
Strain	Mup	EDHB	Mup	EDHB	FIC index	Interpretation
MR196	16	1024	4	128	0.375	Synergy
MR209	16	1024	4	128	0.375	Synergy
MR324	2	512	0.5	256	0.75	Partial synergy
MR366	512	512	64	64	0.25	Synergy
MR490	2	512	0.5	256	0.75	Partial synergy
MR326	8	1024	2	256	0.5	Synergy
MR390	8	1024	4	128	0.625	Partial synergy
MR391	8	1024	2	256	0.5	Synergy
MR398	8	512	4	64	0.625	Partial synergy
MR421	8	1024	2	256	0.5	Synergy
MR422	64	1024	16	64	0.3125	Synergy
MR429	8	1024	2	256	0.5	Synergy
MR444	8	1024	2	128	0.375	Synergy
MR445	8	512	4	64	0.625	Partial synergy
MR454	8	1024	2	256	0.5	Synergy
MR460	8	512	4	32	0.5625	Partial synergy
MR473	8	1024	4	32	0.53125	Partial synergy
MR475	8	512	2	128	0.5	Synergy
MR401	8	1024	2	256	0.5	Synergy
MR404	8	1024	2	128	0.375	Synergy

**Table 3:** Log changes of the viable cells for each treatment during a 24-hour period. .

	ΔLog_10_ cfu/mL between final and initial inoculum						ΔLog_10_ cfu/mL between the combination and the most active drug inoculum	
Strain	Normal control	1/4 MIC Mup	1/2 MIC Mup	EDHB	1/4 MIC Mup+ EDHB	1/2 MIC Mup+ EDHB	1/4 MIC Mup+ EDHB	1/2 MIC Mup+ EDHB
MR196	3.405	3.507	2.804	3.570	2.039	-0.370	-1.468	-3.174
MR209	3.649	3.628	3.422	3.677	1.473	0.247	-2.155	-3.175
MR366	4.059	2.761	2.424	4.066	1.156	-0.098	-1.605	-2.522
MR444	3.822	3.833	3.106	3.863	2.130	-1.029	-1.703	-4.135
MR454	3.889	3.958	3.259	3.739	1.725	0.294	-2.014	-2.965
MR475	3.735	3.911	3.316	3.758	2.929	0.221	-0.829	-3.095

### EDHB disrupts cytoplasmic membrane integrity

**Figure 2:**
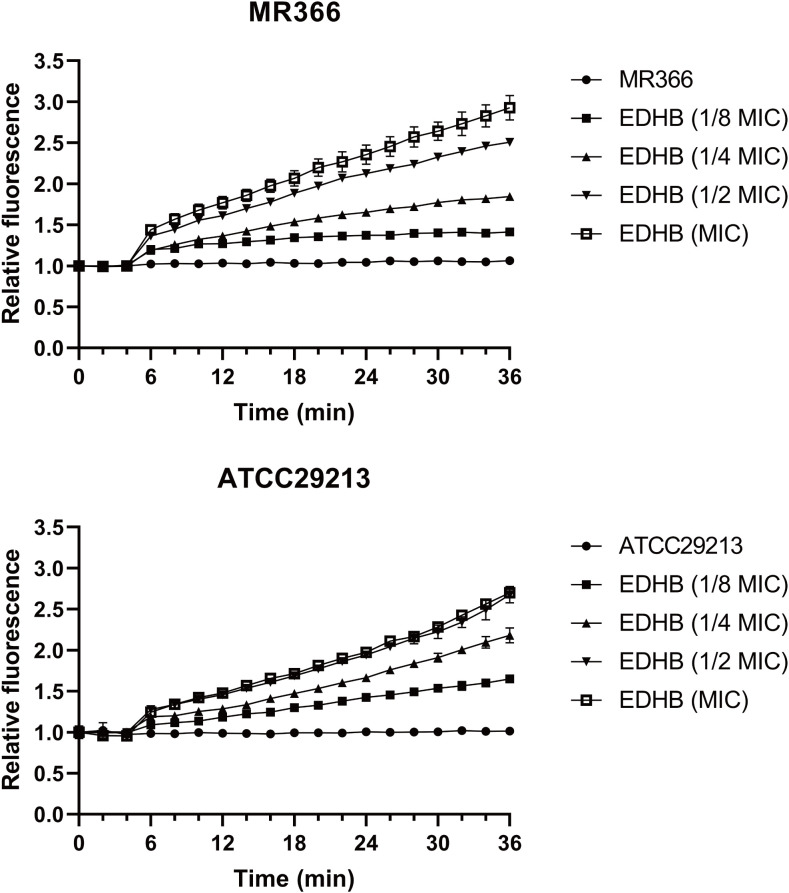
EDHB-induced membrane permeabilization kinetics. Data normalized to untreated controls (n=3).

Cell membrane integrity is critical for bacterial function and viability. PI, which fluoresces upon DNA binding in membrane-compromised cells, exhibited minimal and stable baseline fluorescence in untreated controls, indicating intact cytoplasmic membranes. EDHB treatment induced a concentration-dependent increase in PI fluorescence, demonstrating dose-responsive membrane disruption (**Figure 2**). At the MIC, fluorescence intensity reached nearly threefold higher than controls. This effect occurred rapidly, with significant fluorescence elevation detected within minutes of EDHB exposure. Equivalent membrane permeabilization patterns were observed in both methicillin-sensitive (MSSA) and methicillin-resistant *S. aureus* (MRSA) strains. Collectively, EDHB substantially increased PI uptake across tested strains, as quantified by fluorescence kinetics, confirming its membrane-lytic ability.

### EDHB does not affect *norA* or *mepA* efflux pumps

**Figure 3:**
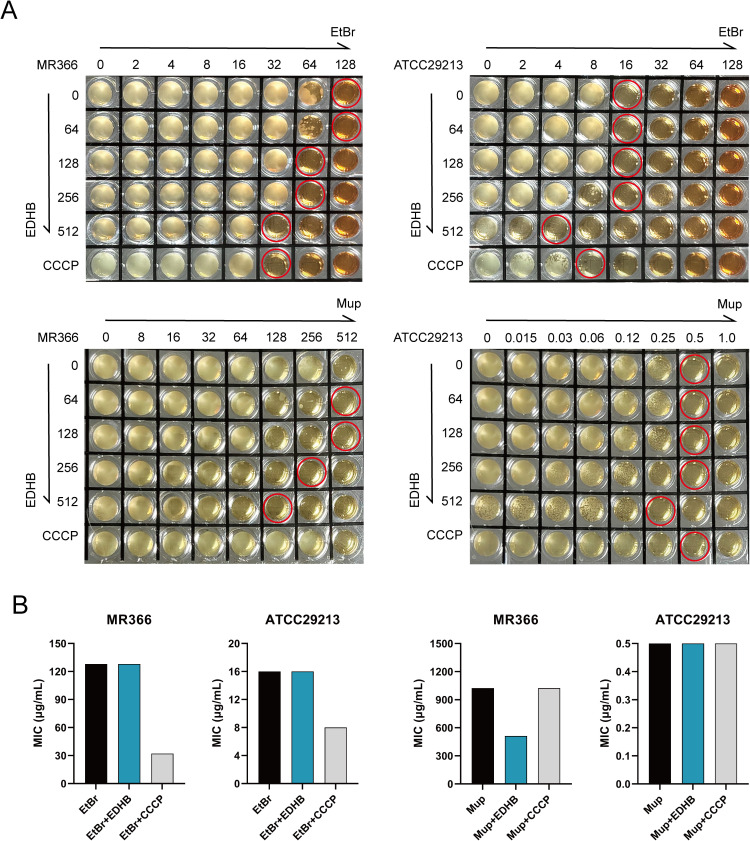
The modification capacity of MIC by the association of EtBr and Mup with EDHB or with the standard inhibitors CCCP against *S. aureus* strains. **(A)** Representative pictures of EtBr or Mup in combination with EDHB or CCCP against MR366 and ATCC29213 using microdilution broth method. **(B)** The MIC values of EtBr and Mup in the presence of 1/8 MIC EDHB or CCCP.

**Figure 4:**
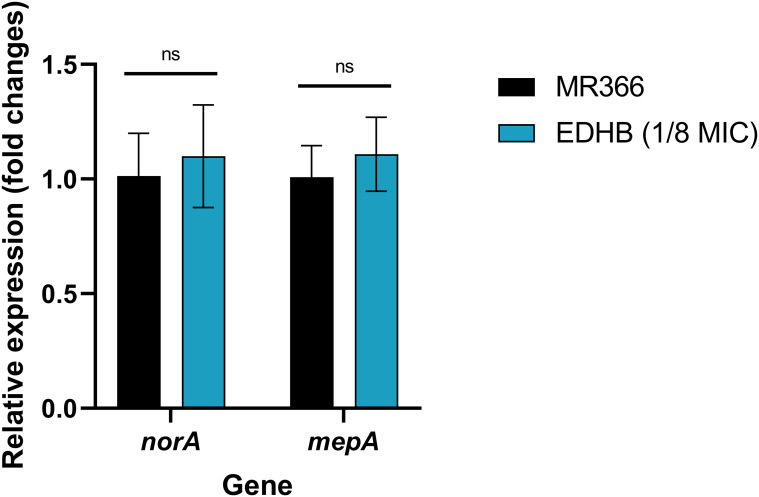
The transcriptional levels of the efflux pump-related genes *norA* and *mepA* in *S. aureus* treated with EDHB. ns, no significance.

Efflux pumps can actively expel EtBr from the intracellular medium, diminishing its cytotoxic accumulation and conferring bacterial survival. EDHB at a subinhibitory concentration (1/8 MIC) failed to synergize with EtBr, as evidenced by unaltered MIC values, though the sedimented bacterial layer of MR366 was moderately reduced at the bottom of the microplate (**Figure 3A**). Synergistic interaction with EtBr emerged only at elevated EDHB concentrations, where growth inhibition mirrored or exceeded that of the efflux pump inhibitor CCCP. EDHB (1/8 MIC) significantly potentiated Mup activity against the resistant strain MR366, reducing its MIC by 1-fold (from 1024 to 512 μg/mL), indicative of resistance reversal. However, no such potentiation occurred in the susceptible strain ATCC29213 (**Figure 3B**). Furthermore, quantitative RT-qPCR revealed no significant alterations in *norA* or *mepA* expression post-EDHB treatment (**Figure 4**), excluding efflux pump transcriptional regulation as a resistance-modifying mechanism.

### Antibacterial efficacy of ointment

**Figure 5:**
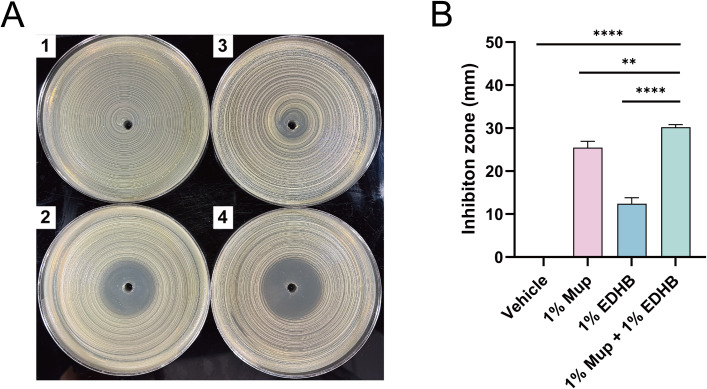
Antibacterial effects of PEG-based ointments toward MR366, a high-level MuR-MRSA strain. **(A)** Inhibition zone of PEG ointments containing 1) vehicle, 2) 1% Mup, 3) 1% EDHB, and 4) 1% Mup + 1% EDHB, and **(B)** the diameter produced by the studied formulations. **, P < 0.01; ****, P < 0.0001.

Given that EDHB potentiates Mup’s antibacterial efficacy through distinct mechanisms of action, we hypothesized that a co-formulated ointment containing both agents might overcome Mup resistance. To preliminarily test this hypothesis, the disk diffusion method was conducted to evaluate the PEG-based ointment’s activity against MR366, a high-level Mup-resistant MRSA. The vehicle control exhibited no growth inhibition against *S. aureus* MR366, while both individual agent and their combination generated distinct zones of inhibition (**Figure 5A**). This confirmed that the ointment matrix does not antagonize either agent’s antimicrobial properties, with the combination producing the largest inhibition zone. Quantitative analysis revealed that 1% Mup and 1% EDHB monotherapies generated inhibition zones of 25.47 mm and 12.44 mm, respectively, whereas their combination achieved a significantly larger inhibition zone of 30.23 mm (P<0.01 vs. either monotherapy) (**Figure 5B**). Significantly, the combination’s enhanced inhibition zone proved that the synergistic effects observed for Mup-EDHB combinations in liquid culture conditions (as previously mentioned) were preserved in topical ointment formulations. These findings further supported the therapeutic potential of this combinatorial strategy against Mup-resistant MRSA.

### Treatment outcome of *S. aureus* wound

**Figure 6:**
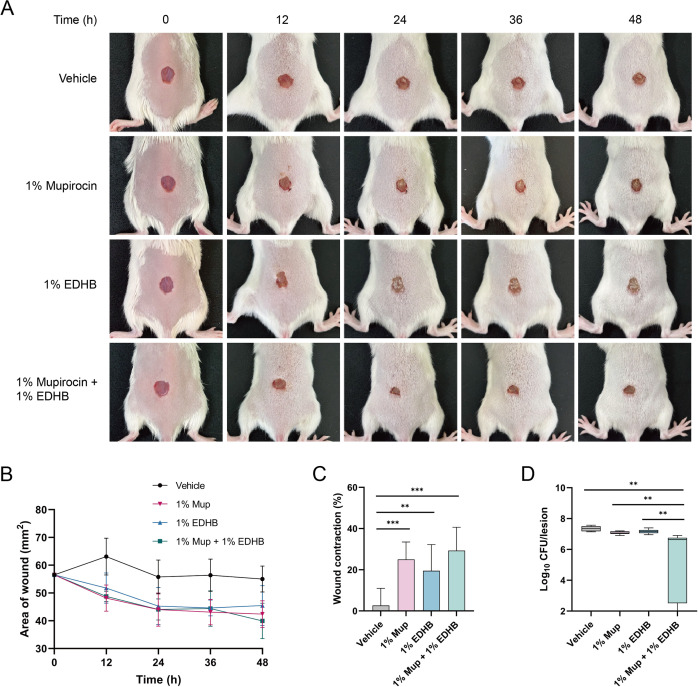
Decolonization treatment in murine wound. **(A)** Representative wound images following 0, 12, 24, 36, and 48 h of treatment with PEG-based ointments containing vehicle, 1% Mup, 1% EDHB, or the combination of 1% Mup and 1% EDHB. **(B)** Average measures of wound area under the same conditions as in panel A, and **(C)** wound contraction at 48 h. **(D)** The number of colonies colonizing the murine wound. **, P < 0.01; ***, P < 0.001.

Therapeutic effects of 1% Mup, 1% EDHB, and their combination in a murine dermal wound model were evaluated. We further investigated whether the drug combination induced significant adverse effects at the wound site. Wound areas were assessed at 12 h intervals, revealing reduced wound contraction across all treatment groups compared to the vehicle control (**Figures 6A, B**). At 48 h, a statistically significant difference was observed between the treatment groups and the vehicle group, though no intergroup differences reached significance among the treatment groups (**Figure 6C**). However, the combination therapy exhibited a trend toward enhanced wound contraction relative to monotherapies, followed by 1% Mup and then 1% EDHB (29.35%; 25.01%; 19.51%; 2.68%). As shown in **Figure 6D**, the results suggested that four applications of 1% Mup reduced the bacterial load by 0.277-log_10_ at the wound site compared to vehicle-treated animals. 1% EDHB monotherapy exhibited minimal efficacy in bacterial clearance. Therefore, neither monotherapy achieved substantial decolonization effects. The combination regimen, however, yielded superior bacterial eradication, reducing wound bacterial load by 2.1-log_10_. Remarkably, only a single MR366 colony was detected in 2 of the 6 animals (33.33%) receiving combination therapy. These findings indicated that the Mup-EDHB co-formulation exhibited enhanced efficacy in reducing *S. aureus* burden at wound sites compared to the individual agents while confirming the capacity to overcome resistance observed with monotherapies.

## Discussion

The rapid spread of antimicrobial resistance (AMR) underscores the critical demand for groundbreaking approaches to address multidrug-resistant bacterial infections. Natural products and their derivatives occupy a pivotal role in pharmaceutical discovery, owing to their unparalleled chemical diversity and potent biological activities (Kumar and Kiran Tudu, 2023). However, they tend to have higher MICs compared to traditional antibiotics, resulting in inadequate therapeutic efficacy when used alone for antibacterial treatment. Interestingly, their combination with antibiotics can markedly suppress the dissemination of drug-resistant bacterial strains. Such combination strategies enhance antibiotic effectiveness by optimizing pharmacokinetic and pharmacodynamic profiles (Caesar and Cech, 2019). In addition, they may lower required drug dosages, thereby mitigating antibiotic-associated adverse effects (Miklasińska et al., 2015). The use of antibiotic adjuvants has been somewhat effective in overcoming multidrug-resistant infections. In this study, the synergistic interaction between Mup and EDHB against MuR-MRSA reveals a promising strategy for overcoming resistance mechanisms. The combination therapy significantly reduced the MICs of both agents (4- to 8-fold reduction), suggesting potential utility in minimizing therapeutic dosages and mitigating concentration-dependent toxicity. The *in vitro* potentiation of Mup by EDHB was successfully translated into improved *in vivo* efficacy, as demonstrated by the formulation containing a formulation combining Mup and EDHB in a mouse skin infection model. While neither Mup nor EDHB monotherapy achieves complete pathogen eradication in wounds, their use in combination can show a stronger antibacterial effect.

Importantly, EDHB exhibits a favorable safety profile supported by both *in vitro* and preclinical evidence. Cytotoxicity assays have demonstrated negligible effects on mammalian cell viability (0.8 mM for astrocytes; 1 mM for rat skeletal muscle myoblasts and rabbit ventricular myocytes) (Philipp et al., 2006; Chu et al., 2010; Nimker et al., 2015), while acute toxicity studies in rat models reveal no adverse clinical signs at therapeutic doses (≤75 mg/kg) (Singh et al., 2016). The concentrations of EDHB used in this study (64, 128, and 256 μg/mL) were converted to molar concentrations of approximately 0.351, 0.702, and 1.405 mM, respectively, based on its molecular weight of 182.18 g/mol. These concentrations are consistent with or below the cytotoxicity thresholds for mammalian cells reported in the literature (Philipp et al., 2006; Chu et al., 2010; Nimker et al., 2015), where negligible effects on cell viability were observed at 0.8 mM for astrocytes and 1 mM for rat skeletal muscle myoblasts and rabbit ventricular myocytes. The two lower concentrations (0.351 and 0.702 mM) were well within the range of non-cytotoxic doses for all tested mammalian cell types, while the highest concentration (1.405 mM) was slightly above the 1 mM threshold for rat skeletal muscle myoblasts and rabbit ventricular myocytes but remained within the context of acute toxicity studies in rat models showing no adverse clinical signs at therapeutic doses (≤75 mg/kg). Overall, the concentrations of EDHB used in this study were considered safe for the experimental assays. As a consequence, a topical formulation combining Mup and EDHB is unlikely to raise toxicity concerns and may represent a valuable option for treating skin infections. Although *in vitro* and *in vivo* models confirmed substantial synergy, the lack of *in vivo* pharmacokinetic/pharmacodynamic (PK/PD) data restricts definitive conclusions regarding tissue penetration or host-microbiome interactions.

The observed synergy (FICI ≤ 0.5) likely stems from the distinct modes of action of the two agents. Mupirocin, a well-characterized isoleucyl-tRNA synthetase inhibitor, specifically targets bacterial protein synthesis (Ho et al., 2018). Conversely, EDHB, a food-derived phenolic compound, can serve as an EPI that reliably enhances the effectiveness of antibiotics. Efflux pumps constitute a key resistance mechanism in *S. aureus*, with their inhibition demonstrating the potential to reverse multidrug resistance phenotypes (Fluman and Bibi, 2009; Al-Sallami et al., 2023). Therefore, the targeted discovery of efflux pump-interfering compounds represents a validated approach to potentiate conventional antibiotics and solve drug resistance mechanisms (Poole and Lomovskaya, 2006). EPIs comprise both natural and synthetic compounds, with the latter often exhibiting undesirable toxicity concerns (Al-Sallami et al., 2023). Given these considerations, it is crucial to identify and develop EPIs from natural origin, particularly plants (Stavri et al., 2007; Tintino et al., 2017; Lu et al., 2022). EPIs mechanistically function by ATP-binding competition and proton gradient interference, leading to energy depletion (Farhat et al., 2020; Macedo et al., 2022). Studies have found that piperine boosts the activity of Mup by inhibiting the MdeA efflux pump (Mirza et al., 2011). Building upon these findings, we made inferences and found that EDHB enhanced Mup efficacy in *S. aureus* not through efflux pump inhibition. This conclusion was supported by multiple lines of experimental evidence. In contrast to classical EPI (CCCP), EDHB failed to reduce the resistance of *S. aureus* strains to Mup. Furthermore, RT-qPCR analysis revealed no significant changes in the transcription levels of efflux pump-associated genes (*norA* and *mepA*) upon EDHB treatment, ruling out a regulatory role in efflux-mediated resistance.

Consequently, elucidation of EDHB’s alternative antibacterial mechanisms remains imperative. The bacterial cytoplasmic membrane, essential for structural integrity and physiological regulation, is a high-value antibiotic target due to its low mutational propensity compared to protein-based systems (Shen et al., 2024). Membrane integrity assays indicated a dose-dependent increase in membrane permeability following EDHB exposure. This disruption likely allowed better mupirocin penetration into cells, which indicated that the cytoplasmic membrane is the primary target of EDHB in *S. aureus.* These findings aligned with previous reports that phenolic compounds, such as gallic acid derivatives, inhibit the growth of bacteria by destabilizing cell membranes, thereby promoting the influx of antibiotics (Keyvani-Ghamsari et al., 2023). The lipophilic nature of membrane-targeting antibacterial compounds facilitates penetration into the cytoplasmic lipid bilayer, underscoring its potential as a pharmacologically underexplored bacterial vulnerability (Cauz et al., 2019). Notably, protocatechuic acid derivatives, such as protocatechuic acid ethyl ester, exhibit superior antibacterial activity compared to the parent compound, potentially due to enhanced lipophilicity that directly correlates with improved membrane permeability and intracellular accumulation (Choinska et al., 2021).

In conclusion, this study identifies EDHB as a novel adjuvant that restores mupirocin efficacy and improves the performance of mupirocin-based ointments against MRSA. The impressive synergy between Mup and EDHB exemplifies a highly strategic approach to effectively combat AMR by engaging multiple targets. This study has certain limitations that should be acknowledged. Notably, although our results support a membrane-targeting mechanism of action for EDHB, the specific molecular targets within the bacterial membrane remain unidentified. Furthermore, the potential for long-term resistance development in MRSA following repeated exposure to the Mup-EDHB combination was not evaluated. The findings of this study imply that future research is essential to thoroughly uncover the antimicrobial mechanism of action of EDHB and to determine its full potential in the treatment of *S. aureus* infections. Besides, rigorous preclinical optimization, encompassing formulation stability assessments and an in-depth analysis of resistance evolution through sequential passaging, will be crucial for propelling this promising strategy toward practical therapeutic applications.

## Data Availability

The original contributions presented in the study are included in the article/supplementary material. Further inquiries can be directed to the corresponding authors.
